# Supplementary material for: Moving Back to the Parental Home in Times of COVID-19: Consequences for Students’ Life Satisfaction
Source: Int J Environ Res Public Health. 2022 Aug 26;19(17):10659. doi: 10.3390/ijerph191710659 (PMC9518347; doi:10.3390/ijerph191710659)
Supplement: Supplementary file 1 [file ijerph-19-10659-s001.zip › ijerph-1827625-supplementary.pdf]

Supplementary Material for Moving back to the parental home in times of COVID-19: Consequences for students’ life satisfaction

Table S1: Summary of Regression models including interaction terms for age, job loss, and gender

Variable		Model 1	Model 2	Model 3	Model 4
Moving back to parental home	No return to parental home	*ref.*	*ref.*	*ref.*	*ref.*
	Return to parental home	-0.352*	3.235*	-0.291	-0.375*
		(0.173)	(1.461)	(0.210)	(0.188)
Employment situation	No change	*ref.*	*ref.*	*ref.*	*ref.*
	Job loss	-0.142	-0.139	-0.107	-0.141
		(0.142)	(0.142)	(0.158)	(0.142)
Age	Age	-0.191	0.017	-0.184	-0.188
		(0.251)	(0.264)	(0.251)	(0.251)
	Age^2	0.003	-0.001	0.003	0.003
		(0.005)	(0.005)	(0.005)	(0.005)
Gender	Female	*ref.*	*ref.*	*ref.*	*ref.*
	Male	0.082	0.074	0.080	0.058
		(0.167)	(0.167)	(0.167)	(0.184)
Return to parental home x Age			-0.159*		
			(0.064)		
Return to parental home x Job loss				-0.180	
				(0.352)	
Return to parental home x Gender = Male					0.140
					(0.445)
Intercept		10.205**	7.368*	10.103**	10.168**
		(3.120)	(3.317)	(3.128)	(3.124)
R^2		0.011	0.018	0.011	0.011
adjusted R^2		0.006	0.011	0.005	0.005
F		2.044	2.732*	1.746	1.719
N		913	913	913	913

Note. *p < .05. **p < .01. ***p < .001. Standard errors in parentheses.
